# Fatigue Life Prediction of 2024-T3 Al Alloy by Integrating Particle Swarm Optimization—Extreme Gradient Boosting and Physical Model

**DOI:** 10.3390/ma17215332

**Published:** 2024-10-31

**Authors:** Zhaoji Li, Haitao Yue, Ce Zhang, Weibing Dai, Chenguang Guo, Qiang Li, Jianzhuo Zhang

**Affiliations:** School of Mechanical Engineering, Liaoning Technical University, Fuxin 123000, China

**Keywords:** physical model, multi-parameter, Al alloy, particle swarm optimization, extreme gradient boosting, fatigue life

## Abstract

The multi-parameter characteristics of the physical model pose a challenge to the fatigue life prediction of 2024-T3 aluminum (Al) alloy. In response to this issue, a parameter-solving method that integrates particle swarm optimization (PSO) with extreme gradient boosting (XGBoost) is proposed in this study. The fatigue performance and failure mechanism of the 2024-T3 Al alloy are analyzed. Furthermore, the fatigue life prediction physical model of the 2024-T3 Al alloy is established by using the energy method of fracture mechanics. The physical model incorporates critical physical parameters. Meanwhile, the PSO algorithm optimizes the hyperparameters of the XGBoost model based on fatigue data of the 2024-T3 Al alloy. Eventually, the optimized XGBoost model is used to solve the parameters of the physical model. Furthermore, the analytical equation of the fatigue life prediction model is obtained. This paper provides a new method for solving the parameters of the fatigue life prediction model, which reduces the error and cost of obtaining the model parameters in the experiment and shortens the time required.

## 1. Introduction

Due to their excellent strength-to-weight ratio and corrosion resistance, Al alloys are widely used in the aerospace, automotive, and construction industries [1,2]. However, Al alloy structural components have been subjected to alternating loads, presenting a risk of fatigue fractures [3]. This poses a threat to the safety of transport equipment. In particular, a fatigue life prediction model is of great significance for the fatigue reliability design of Al alloy structural parts. However, the construction of a fatigue life prediction model faces several challenges, including the complexity of the material’s microstructure, the diversity of manufacturing processes, and variations in actual service environments [4]. Zhou [5] et al. and Nowell D [6] et al. demonstrated that microstructural defects in aluminum alloys, such as pores and inclusions, significantly impact the initiation and propagation of fatigue cracks. This leads to challenges in predicting fatigue life. Additionally, the anisotropy of Al alloys and the mechanical property variations resulting from different heat treatment histories make fatigue life prediction more complex [7]. The fatigue performance is also significantly impacted by environmental factors, such as temperature, humidity, and corrosive media, further complicating the prediction [8]. Therefore, predicting the fatigue life of Al alloys requires a comprehensive consideration of multiple factors, posing significant challenges to improving prediction accuracy and reliability.

In recent years, significant progress has been made in predicting the fatigue life of Al alloys using traditional physical material research methods. Researchers have conducted experimental tests and analytical modeling to deeply investigate the behavior of Al alloys under cyclic loading, thereby enhancing the accuracy of fatigue life prediction [9]. Chen et al. [10] proposed a fatigue life prediction of a 6061-T6 Al alloy based on defect analysis. However, the constructed life prediction model did not deeply consider the fatigue failure mechanism. Chabouk et al. [11] used the Manson–Coffin–Basquin equation to estimate the fatigue life of a 2024-T351 Al alloy. Due to the inability to obtain analytical solutions for the life prediction equation, the application of the model is limited. Moreover, the lack of the fatigue failure mechanism analysis results in the poor interpretability of the life prediction model. Consequently, the fatigue life prediction models in the above studies have not fully integrated the impact of microstructural defects and their evolution on fatigue behavior. Therefore, the accuracy and applicability of the life predictions made are limited. Cauthen et al. [12] studied the microstructural fatigue crack growth behavior of AA7065 and AA2099 Al alloys. The results show that crack initiation in the AA7065 is mainly due to voids and intermetallic particles, while persistent slip bands and intermetallic particles lead to the fatigue failure of AA2099. Wisner et al. [13] examined the impact of specimen geometry and loading schemes on particle fracture in Al alloys. Hence, the understanding of fatigue crack initiation is enhanced. This provides a theoretical basis for establishing a fatigue life prediction model. However, the fatigue life prediction model based on physical models contains multiple parameters. The parameters require extensive experimental data for parameter fitting [14]. Notably, it takes a lot of time to obtain the model parameters. Moreover, the parameter values depend on the accuracy of the test equipment and the data processing method. The explainable fatigue life prediction physical equation and its parameter-solving method are two important issues in realizing the accurate prediction of fatigue life.

Advanced machine learning methods demonstrate significant advantages in handling high-dimensional data and complex nonlinear relationships. Specifically, based on elastoplastic fatigue damage and machine learning models, Zhan et al. [15] proposed a novel approach for predicting the fatigue life of aerospace alloy components. The results showed that this method outperformed traditional approaches in terms of predictive accuracy and generalizability. In addition, Pałczy et al. [16] demonstrated the successful application of machine learning techniques in multiaxial fatigue life prediction, proving that these techniques are highly adaptable under complex multiaxial loading conditions. Furthermore, Raja et al. [17] confirmed the effectiveness of machine learning in predicting fatigue crack growth behavior in aluminum alloys, especially under high-stress and complex-stress conditions. Methods such as Support Vector Machine (SVM), Random Forest (RF), and Extreme Gradient Boosting (XGBoost) exhibit superior performance in handling high-dimensional data and capturing complex relationships within the data [18,19]. These techniques utilize large datasets to identify patterns and make predictions, significantly enhancing the capabilities beyond traditional empirical methods. Despite the potential of machine learning, data-driven models alone have limitations [20,21]. These models typically lack physical interpretability and may not reveal the underlying fatigue failure mechanism of specimens [9,22]. In addition, the predictive performance of machine learning models is highly dependent on the quality and quantity of the training data. Moreover, the results are affected by data noise and imbalance [23]. Thus, the key to improving the accuracy of fatigue life prediction is to study the fatigue life prediction method by combining machine learning and physical models.

In this study, integrating machine learning with physical modeling is investigated to address the issues in the construction of traditional fatigue life prediction models. This hybrid approach aims to leverage the advantages of both methodologies, providing accurate predictions along with physically meaningful insights [24]. By incorporating physical principles into the machine learning framework, these models can offer better generalization and robustness, particularly in scenarios with limited data [25]. This approach not only improves the predictive performance but also enhances the model’s transparency, making it more acceptable for practical engineering applications [26]. Foremost, the tension–tension fatigue life of the 2024-T3 Al alloy is tested under high and low cycle fatigue conditions. The fatigue failure mechanism of the specimen is analyzed. Furthermore, based on crack propagation theory and energy release theory, a physical model for predicting the fatigue life of the Al alloy is proposed. According to the fatigue life data, PSO is used to optimize the hyperparameters of the XGBoost model, enhancing its predictive accuracy and robustness. Finally, the parameters of the physical model are determined by the fatigue life predictions from the optimized machine learning model. By integrating machine learning with a physical model, this study presents a comprehensive fatigue life prediction model for the Al alloys. The model determines the precise values of parameters within the physical model based on prediction from machine learning, offering not only accurate predictions but also physical interpretations, thereby meeting the complex requirements of practical applications.

## 2. Theoretical Framework and Materials

### 2.1. Material and Experiments

The experimental material used in this study is 2024-T3 bare Al alloy (Ra = 0.8 µm). The Al alloy is provided by Shenyang Aerospace University, and the size of each Al alloy plate is 915 mm × 380 mm × 1.6 mm. The chemical composition and mechanical properties of the Al alloy are shown in Table 1 and Table 2, respectively. The results come from our previous studies [27].

**Figure 1:**
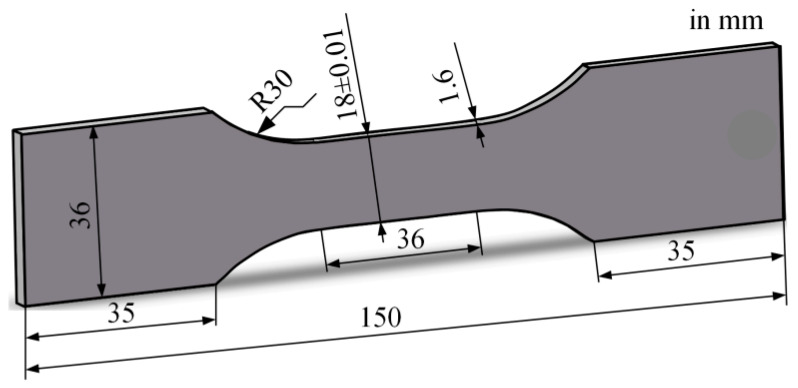
Geometry of the fatigue specimens.

For the preparation of fatigue specimens, wire electrical discharge machining was used to prevent deformation of the plate. The geometric size of the specimens is shown in Figure 1. In addition, the specimens obtained by wire-cutting were ultrasonically cleaned in deionized water in time to avoid surface corrosion of the cutting fluid, followed by drying. After the workpieces were cut from the plates, their wire-cutting surface was polished step by step with 400-2000# sandpaper. Then, the specimens were cleaned with alcohol using an ultrasonic cleaner, followed by drying with a hair dryer.

**Figure 2:**
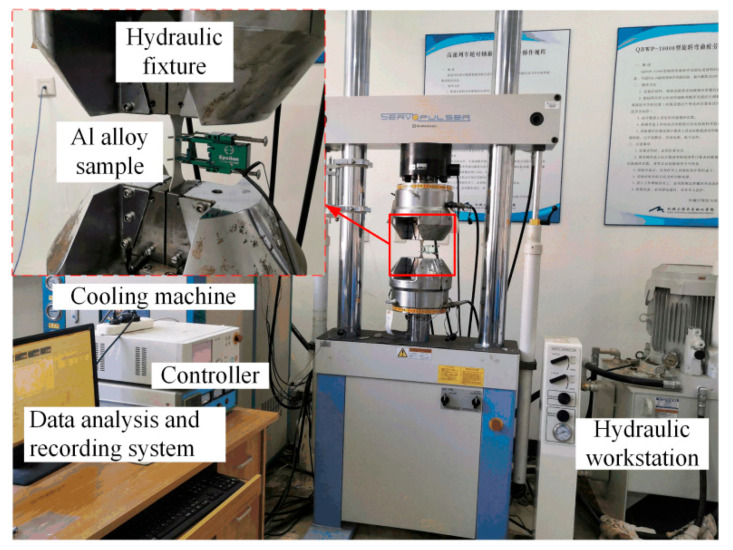
Fatigue test machine in this study.

**Figure 3:**
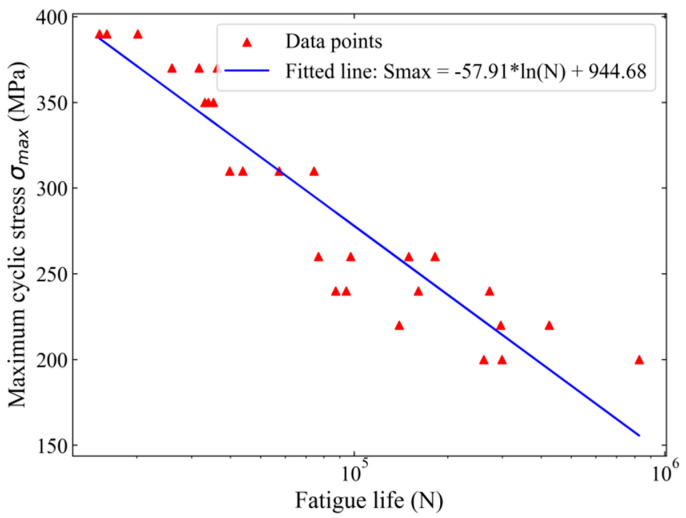
Fatigue life under the eight stress levels.

The fatigue tests were conducted using an electro-hydraulic fatigue testing machine (EHF-EV200K2-040-1A, Shimadzu, Kyoto, Japan). The fatigue testing machine primarily consists of a controller, a hydraulic workstation, hydraulic fixtures, and a cooling machine, as shown in Figure 2. All fatigue test data were recorded by a data analysis and recording system when fatigue failure occurred in the Al alloy specimens. The setting parameters for fatigue life assessment are a sinusoidal waveform, loading force, a stress ratio R of 0.1, and a frequency of 20 Hz. The fatigue life was obtained under the maximum cyclic stresses σmax of 200, 220, 240, 260, 310, 350, 370, and 390 MPa. Under each stress level, from three to four parallel tests were carried out. The results are shown in Figure 3. The statistical analysis of the fatigue life data is presented in Table 3.

### 2.2. Overview of Fatigue Mechanics and Physical Models

**Figure 4:**
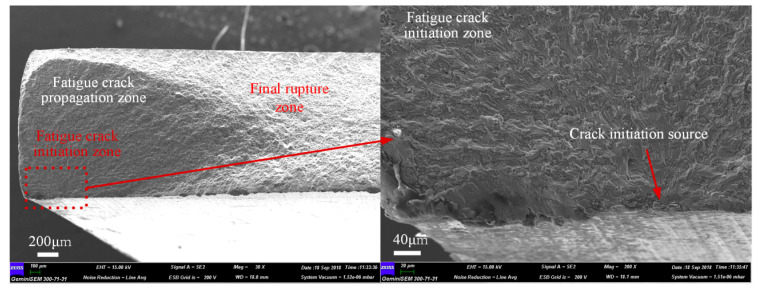
Fatigue fracture of the 2024-T3 Al alloy.

**Figure 5:**
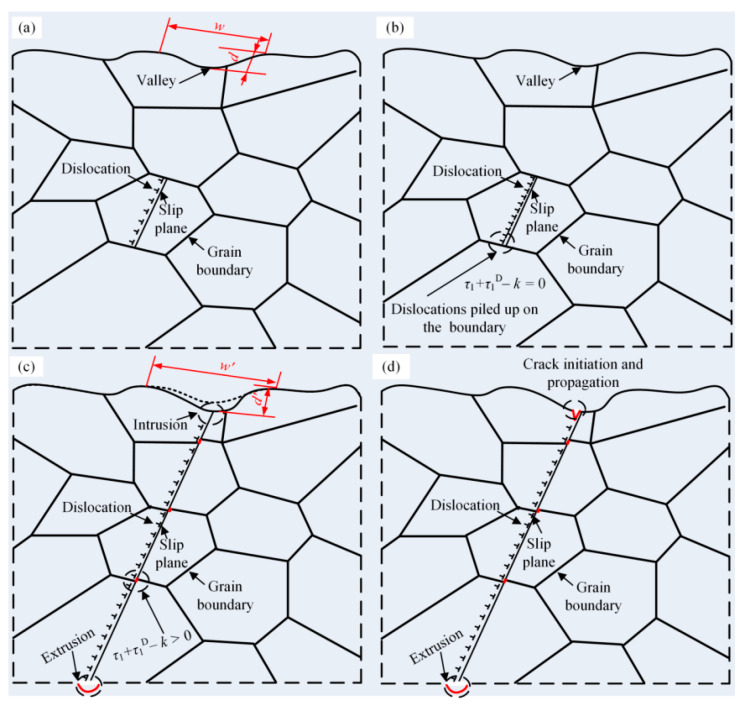
Schematic diagram of fatigue failure mechanism of the 2024-T3 Al alloy based on dislocation slip.

To reveal the fatigue failure mechanism of the 2024-T3 Al alloy is an important problem in establishing the fatigue life prediction model [28]. Figure 4 shows a scanning electron microscope (SEM) image of the 2024-T3 Al alloy undergoing fatigue fracture at the maximum cyclic stress of 220 MPa. The results indicate that a fatigue crack was initiated at the specimen surface, and the fatigue crack extended approximately 45 degrees inside the Al alloy. For the specimens subjected to tensile stress, the maximum shear stress *τ*_1_ is in the 45-degree direction. Since the roughness of the 2024-T3 Al alloy is 0.8, there are peaks and valleys on its surface, as shown in Figure 5a. This results in local stress concentration on the specimen surface. The stress concentration coefficient *k_t_* is related to the depth *d* of the valley [29], as shown in Equation (1).

(1) $$k_{t}=1+\sqrt{\frac{d}{\rho }}$$

where *ρ* is valley root radius. The increase in the surface roughness increased the *k_t_*. In addition, stress intensity factor Δ*K* is another important parameter for evaluating fatigue performance. The Δ*K* is expressed as follows [30]:

(2) $$\Delta K=Y\sigma \sqrt{\pi a}$$

where *Y* is a shape factor, *σ* is the amplitude of the stress, and *a* is the crack length. The increase in the *k_t_* increased the Δ*K*. Thus, the fatigue cracks with high roughness are easy to form on the surface, and fatigue life is poor [31].

In addition to the surface roughness, the intrusion caused by dislocation slip also leads to the stress concentration on the surface of the 2024-T3 Al alloy. As shown in Figure 5b, the equilibrium condition of dislocations can be expressed as follows:

(3) $$\tau_{1}^{D}+\tau_{1}-k=0$$

where *τ*_1_*^D^* is the back stress due to dislocations piled up on the boundary and *k* is the frictional stress. Due to the free surface of the specimen, a minor hindrance induces the dislocations to slip towards the surface, as shown in Figure 5c. Because *k*, grain size 2*a*′, Burgers vector *b*, and *A*, related to the dislocation stress field, are determined by material type, the number of dislocations *N_D_* of the 2024-T3 Al alloy depends on the shear stress ($N_{D}=(\tau_{1}-k)a^{\prime }/\pi A$) [32].

The large shear stress *τ*_1_ enables dislocations to break through the obstacles of grain boundaries, and a large number of dislocations gradually slip toward the surface of the specimen. The dislocation slip induces intrusion and extrusion zones on the surface of the specimen, as shown in Figure 5c. The size of the intrusion and extrusion zones is determined by the plastic displacement *γ*_1_ ($\gamma_{1}=\pi N_{D}ba^{\prime }/2$). The increase in ND results in the large *γ*_1_. Consequently, the size of the intrusion dIn increases at the direction of maximum shear stress *τ*_1_. For specimens subjected to certain cyclic stress, the valley depth d’ is positively correlated with dIn (dIn = d’ − d). This implies that the stress concentration generated by the extrusion zone and valley induces cracks on the surface of the 2024-T3 Al alloy specimen, as shown in Figure 5d. The fatigue failure mechanism revealed in Figure 4 is consistent with the failure characteristics exhibited by the fracture surface.

Tanaka et al. pointed out that dislocation slip induces crack initiation and propagation accompanied by energy release [33]. Moreover, fatigue failure primarily resulted from the growth of microstructural defects under cyclic loading [34]. The energy release rate, denoted as *G*, quantifies the energy dissipated per unit area of the new crack surface formed. Moreover, *G* is a pivotal factor in understanding crack propagation dynamics [35]. The rate of energy release is intricately linked to the stress intensity factor range Δ*K* at the crack tip, which reflects the localized stress state influencing crack growth. This relationship between the *G* and Δ*K* is expressed as follows:

(4) $$G=\frac{{\left(\Delta K\right)}^{2}}{E^{\prime }}$$

where *E*′ represents the effective modulus of elasticity. The precise calculation of *G* facilitates the estimation of how quickly a crack may propagate through the Al alloy, thus directly affecting the fatigue life.

Paris’ law provides the relationship between the crack propagation rate and the cyclic stress [36]. The equation is as follows:

(5) $$\frac{da}{dN}=C{\left(\Delta K\right)}^{m}$$

where *N* denotes the number of stress cycles the specimen is subjected to. *C* and *m* are material constants. Equation (4) can be rewritten as follows:

(6) $$\Delta K=\sqrt{GE^{\prime }}$$

Substituting Equation (6) into Equation (5), the crack growth rate equation is defined as follows:

(7) $$\frac{da}{dN}=C{\left(GE^{\prime }\right)}^{m/2}$$

This equation demonstrates the response of the Al alloy to cyclic loading, where *C* and *m* indicate the sensitivity of crack growth rate to the cyclic stresses.

As shown in Figure 3 and Figure 4, the fatigue failure of the 2024-T3 Al alloy is attributed to the formation and propagation of the cracks. Thus, the fatigue failure process can be modeled dynamically by defining a state vector *x*(*t*) and an input vector *u*(*t*). Their functions are, respectively, as follows:

(8) $$x(t)=\left[\begin{array}{c} a(t)\\ G(t) \end{array}\right]$$

and

(9) u(t)=σ(t)

where *a*(*t*) represents the dynamic crack length. *G*(*t*) denotes the dynamic energy release rate. *σ*(*t*) is the dynamic stress amplitude. The state–space model exhibits the evolution of crack size and energy release over time. Thus, Equation (6) can be expressed as follows:

(10) $$a(t)=C{\left(G(t)E^{\prime }\right)}^{m/2}$$

According to Equations (2) and (6), *G*(*t*) is expressed as follows:

(11) $$G(t)=\frac{Y\sigma (t)}{E^{\prime }\sqrt{\pi a(t)}}$$

where *Y* reflects the influence of the crack’s shape and loading mode on its propagation. a(t) represents the dynamically change rate of crack length and G(t) denotes the dynamically change rate of energy release. This state–space model exhibits the temporal evolution of crack size and energy release, which is essential for simulating the fatigue behavior of the Al alloy.

Notably, the crack length had a critical size during the propagation process, which is called the critical crack length ac. The parameter ac refers to the maximum allowable crack length before the specimen enters an unstable fracture state. When the crack reaches ac, the crack is at its critical state, and any further growth could result in the rapid propagation and failure of the specimen. In addition, when the stress intensity factor ΔK of the Al alloy reaches its fracture toughness, $\Delta K_{IC}$, the specimen also enters an unstable fracture. Using the relationship between the stress intensity factor and crack length (Equation (2)), the relationship between ac and $\Delta K_{IC}$ is expressed as follows:

(12) $$a_{c}={\left(\frac{\Delta K_{IC}}{Y\sigma \sqrt{\pi }}\right)}^{2}$$

Li et al. indicated that the initial crack length *a*_0_ was proportional to the surface roughness Ra. Moreover, the quantitative relationship between the Ra of the specimen and the *a*_0_ is [37] as follows:

(13) $$a_{0}=2.97\mathrm{Ra}$$

Based on the analysis of dynamic characteristics of the fatigue crack growth, the fatigue life *N_f_* of the Al alloy is as follows:

(14) $$N_{f}=\int_{a_{0}}^{a_{c}}\frac{1}{C{\left(\sqrt{GE^{\prime }}\right)}^{m}}da$$

Substituting Equations (12) and (13) into Equation (14), the fatigue life calculation equation for the 2024-T3 Al alloy is obtained as follows:

(15) $$N_{f}=\frac{2}{C{\left(Y^{2}\sigma^{2}\pi \right)}^{m/2}(m-2)}\left[{\left(\frac{\Delta K_{IC}}{Y\sigma \sqrt{\pi }}\right)}^{2(1-m/2)}-{\left(2.97\mathrm{Ra}\right)}^{1-m/2}\right]$$

## 3. Methodology: Integrating Machine Learning with Physical Model

### 3.1. Machine Learning Model

#### 3.1.1. Support Vector Machine

Support vector machine (SVM) aims to identify a hyperplane that minimizes the distance of all specimen points from this plane. SVM is particularly advantageous in nonlinear mapping and high-dimensional pattern recognition [38]. Notably, the output of SVM is a continuous value. Moreover, the ε-insensitive loss function is integrated into SVM to facilitate regression analysis, termed Support Vector Regression [39]. The analysis of the fatigue failure of the 2024-T3 Al alloy shows that cracking was a process of continuous change accompanied by changes in energy. For the fatigue life prediction, SVM is an advantageous tool for solving equation parameters. In linear regression analysis, the goal is to derive a regression model ($f(x)=\omega^{T}\cdot x+b$) that ensures each training specimen is as close as possible to *f*(*x*). The maximum allowable error between the specimen and the model *f*(*x*) is designated as *ε*. Loss is computed when $|f(x_{j})-y_{j}|>\varepsilon$. The *ε*-insensitive loss function is defined as follows:

(16) $$l_{\varepsilon }=\left\{\begin{array}{ll} 0, & |f(x_{j})-y_{j}|<\varepsilon \\ |f(x_{j})-y_{j}|-\varepsilon , & \mathrm{other} \end{array}\right.$$

Based on the maximum margin optimization principle of SVM, the optimization objective for regression analysis can be formulated as follows:

(17) $$\min\frac{1}{2}∥\omega {∥}^{2}+C_{i}\sum_{i=1}^{n}l_{\varepsilon }(f(x_{j})-y_{j})$$

where *C_i_* is the regularization parameter. To solve this optimization problem, non-negative slack variables $\xi_{j}$ and ${\hat{\xi }}_{j}$ are introduced, leading to the following reformulated objective:

(18) $$\min\frac{1}{2}∥\omega {∥}^{2}+C_{i}\sum_{i=1}^{n}\left(\xi_{j}+{\hat{\xi }}_{j}\right)$$

Equation (18) yields the following:

(19) $$\left\{\begin{array}{l} \omega^{T}\cdot x_{j}+b-y_{j}\leq \varepsilon +\xi_{j}\\ y_{j}-\omega^{T}\cdot x_{j}-b\leq \varepsilon +{\hat{\xi }}_{j}\\ \xi_{j}\geq 0,\;{\hat{\xi }}_{j}\geq 0,\;j=1,2,\ldots ,n \end{array}\right.$$

In order to convert this constrained optimization problem into an unconstrained one, the Lagrangian function is introduced to Equation (18). The goal function is expressed as follows:

(20) $$\begin{array}{c} L=\frac{1}{2}∥\omega {∥}^{2}+C\sum_{j=1}^{n}\left(\xi_{j}+{\hat{\xi }}_{j}\right)-\sum_{i=1}^{n}\left(u_{j}\xi_{j}+{\hat{u}}_{j}{\hat{\xi }}_{j}\right)\\ -\sum_{j=1}^{n}\alpha_{j}(\xi_{j}+{\hat{\xi }}_{j}-y_{j}+\omega^{T}\cdot x+b)-\sum_{j=1}^{n}{\hat{\alpha }}_{j}(\xi_{j}+{\hat{\xi }}_{j}+y_{j}-\omega^{T}\cdot x-b) \end{array}$$

where $u_{j}\geq 0$, ${\hat{u}}_{j}\geq 0$, $\alpha_{j}\geq 0$ and ${\hat{\alpha }}_{j}\geq 0$ are Lagrange multipliers. By taking partial derivatives with respect to ω, *b*, $\xi_{j}$, and ${\hat{\xi }}_{j}$, the following equations can be derived.

(21) $$\sum_{j=1}^{n}\left({\hat{\alpha }}_{j}-\alpha_{j}\right)x_{j}=\omega$$

(22) $$\sum_{j=1}^{n}\left({\hat{\alpha }}_{j}-\alpha_{j}\right)=0$$

(23) $$\alpha_{j}+u_{j}=C_{i}$$

(24) $${\hat{\alpha }}_{j}+{\hat{u}}_{j}=C_{i}$$

Substituting Equations (21)–(24) into Equation (20), the dual problem of SVR can be obtained. The expression is as follows:

(25) $$\max\sum_{j=1}^{n}y_{j}({\hat{\alpha }}_{j}-\alpha_{j})-\varepsilon \sum_{j=1}^{n}\left({\hat{\alpha }}_{j}+\alpha_{j}\right)-\frac{1}{2}\sum_{j,k=1}^{n}\left({\hat{\alpha }}_{j}-\alpha_{j})({\hat{\alpha }}_{k}-\alpha_{k}\right)x_{k}^{T}\cdot x_{k}$$

Moreover, Equation (25) satisfies the Karush-Kuhn-Tucker conditions. The equations are as follows:

(26) $$\sum_{j=1}^{n}\left({\hat{\alpha }}_{j}-\alpha_{j}\right)=0$$

(27) $$0\leq {\hat{\alpha }}_{j},\alpha_{j}\leq C_{i}$$

Inserting the solution into the linear regression model, the prediction function is as follows:

(28) $$f(x)=\sum_{i=1}^{n}\left({\hat{\alpha }}_{i}-\alpha_{i}\right)x_{i}^{T}x+y_{i}+\varepsilon -\sum_{j=1}^{n}\left({\hat{\alpha }}_{j}-\alpha_{j}\right)x_{j}^{T}x_{i}$$

For nonlinear regression analysis, by incorporating the kernel function *f*(*x_i_*,*x*), the prediction function is modified to the following:

(29) $$f(x)=\sum_{j=1}^{n}\left({\hat{\alpha }}_{j}-\alpha_{j})k(x_{j},x\right)+y_{i}+\varepsilon -\sum_{k=1}^{n}\left({\hat{\alpha }}_{k}-\alpha_{k})k(x_{j},x_{k}\right)$$

In this study, the radial basis function (RBF) kernel is utilized due to its effectiveness in extending features to infinite dimensions and its ease of parameter tuning.

#### 3.1.2. Random Forest

Random Forest (RF) is an ensemble learning method that enhances prediction accuracy and robustness by constructing multiple decision trees and aggregating their results [40]. RF exhibits significant advantages in handling high-dimensional data and nonlinear problems. For predicting fatigue life, RF is employed in regression analysis.

In regression analysis, the objective is to build a model *f*(*x*). The model approximates the training samples (*x_l_*,*y_l_*) as closely as possible. Randomness is introduced by generating multiple decision trees for RF. This decreases the overfitting and improves generalization. Each decision tree consists of a random sample set (obtained via bootstrapping from the training data) and a random subset of features. Each decision tree in the forest predicts using the mean squared error (MSE) as the loss function, defined as follows:

(30) $$MSE=\frac{1}{n}\sum_{l=1}^{n}{\left(y_{l}-{\hat{y}}_{l}\right)}^{2}$$

where *y_l_* represents the actual value and ${\hat{y}}_{l}$ denotes the predicted value. The RF prediction is determined by averaging the predictions from all the trees. The optimization objective of RF can be expressed as follows:

(31) $$\min\frac{1}{n}\sum_{l=1}^{n}{\left(y_{l}-\frac{1}{T}\sum_{t=1}^{T}f_{t}(x_{l})\right)}^{2}$$

where *T* is the number of decision trees, and *f_t_*(*x_l_*) is the prediction of the *t*-th tree for sample *x_l_*.

To avoid overfitting, RF applies several regularization techniques. These include limiting the tree depth, setting a threshold for node splits, and specifying a minimum number of samples for leaf nodes. In order to convert this constrained optimization problem into an unconstrained one, the Lagrangian function is introduced to Equation (32). The goal function is expressed as follows:

(32) $$L=\frac{1}{2}∥\theta_{1}{∥}^{2}+C_{j}\sum_{l=1}^{n}{\left(y_{l}-{\hat{y}}_{l}\right)}^{2}$$

where *θ*_1_ represents the model parameters, *C_j_* is the regularization coefficient and ${\hat{y}}_{l}$ is the predicted value for sample *x_l_*. To minimize this function, we set the partial derivatives as follows with respect to *θ*_1_ to zero:

(33) $$\frac{\partial L}{\partial \theta_{1}}=0$$

The prediction process of RF is as follows: Firstly, each decision tree independently predicts the output for a given input sample. Secondly, the final prediction is obtained by averaging the results from all the trees. In practice, cross-validation techniques are employed to evaluate the performance of the RF model, and grid search is used to optimize hyperparameters such as the number of trees, maximum depth, and minimum samples required for a split. The optimization goal in each tree node is to maximize the information gain or minimize the mean squared error, expressed as follows:

(34) $$\max\sum_{i=1}^{n}I(G_{i})$$

where *I*(*G_i_*) represent the information gain at node *G_i_*.

In this study, RF was selected due to its robust performance in handling high-dimensional and nonlinear data, along with its high prediction accuracy and stability. By appropriately tuning parameters and optimizing the model, RF can effectively predict fatigue life and provide reliable regression analysis results.

#### 3.1.3. Extreme Gradient Boosting

Extreme Gradient Boosting (XGBoost) is an advanced ensemble learning technique that employs gradient boosting algorithms to enhance prediction accuracy and robustness [41]. XGBoost is highly effective in handling high-dimensional data and complex nonlinear relationships. For predicting fatigue life, XGBoost is employed in regression analysis.

In regression analysis, the objective is to build a model *f*(*x*) that approximates the training samples (*x_p_*, *y_p_*) as closely as possible. XGBoost constructs an ensemble of decision trees, where each tree is sequentially added to correct the errors made by the previous trees. The loss function used in XGBoost for regression is typically the mean squared error (MSE). The optimization objective of XGBoost combines the loss function with a regularization term to avoid overfitting, expressed as follows:

(35) $$\min\sum_{p=1}^{n}l(y_{p},{\hat{y}}_{p})+\sum_{k=1}^{K}\Omega (f_{k})$$

where *l* is the loss function. $\Omega (f_{k})=\gamma T+\frac{1}{2}\lambda ∥{w∥}^{2}$ is the regularization term for the *k*-th tree. *w* is the number of leaves in the tree. *γ* and *λ* are regularization parameters.

The training process of XGBoost involves the following steps. The model is initialized with a base prediction, which is typically set as the mean of the target values. In each iteration, the gradient and hessian of the loss function with respect to the current predictions are computed. A decision tree is then fitted to the negative gradients (also known as residuals), with the Hessian being used to weight the gradients. The model is updated by adding the predictions from the newly fitted tree. Regularization techniques are applied to control the complexity of the model, ensuring better generalization and avoiding overfitting.

The objective function in each iteration is given by the following:

(36) $$L^{\left(t\right)}=\sum_{n}^{i=1}[g_{i}f_{t}(x_{p})+\frac{1}{2}h_{i}f_{t}{\left(x_{p}\right)}^{2}]+\Omega (f_{t})$$

where *g_i_* and *h_i_* are the gradient and hessian of the loss function, respectively. In order to convert this constrained optimization problem into an unconstrained one, the Lagrangian function is introduced to Equation (36). The goal function is expressed as follows:

(37) $$L=\sum_{n}^{i=1}l(y_{p},{\hat{y}}_{p})+\sum_{K}^{k=1}\Omega (f_{k})+\sum_{m}^{j=1}\lambda_{j}\left(\sum_{n}^{i=1}w_{ij}-\theta_{j}\right)$$

where *λ_j_* is the Lagrange multipliers, *w_ij_* is the weights, and *θ_j_* is the constraints. By taking the partial derivatives with respect to the model parameters and setting them to zero, the optimal solution can be derived as follows:

(38) $$\frac{\partial L}{\partial \theta_{j}}=0$$

In practice, XGBoost uses additional techniques such as shrinkage (learning rate), column subsampling, and early stopping to improve model performance. Moreover, overfitting issues are avoided. The prediction function of XGBoost can be expressed as follows:

(39) $$\hat{y}=\sum_{k=1}^{K}f_{k}(x)$$

where *K* is the total number of trees. *f_k_* is the prediction from the *k*-th tree. Advantages of XGBoost include its scalability, efficiency, and ability to handle missing values.

In this study, XGBoost was selected due to its superior performance in handling high-dimensional and nonlinear data, along with its high prediction accuracy and robustness. By appropriately tuning parameters and optimizing the model, XGBoost can effectively predict fatigue life and provide reliable regression analysis results.

### 3.2. Particle Swarm Optimization

Particle Swarm Optimization (PSO) is a swarm intelligence-based optimization algorithm that mimics the foraging behavior of birds to search for the optimal solution [42]. PSO demonstrates significant advantages in optimizing complex high-dimensional functions and multimodal problems [43]. In this study, PSO is employed to optimize the parameters of machine learning models to enhance prediction accuracy and robustness.

A swarm of particles moves through the search space to find the optimal solution. This is the central idea of the PSO algorithm. Each particle represents a candidate solution with attributes of position and velocity. The movement of particles is influenced by both their own experience and the experience of the swarm. The objective of PSO is to minimize or maximize an objective function, which, in this study, is the prediction error of the machine learning model. The position and velocity update formulas for particles are as follows:

(40) $$v_{i}(t+1)=\omega v_{i}(t)+c_{1}r_{1}(p_{i}-x_{i}(t))+c_{2}r_{2}(g-x_{i}(t))$$

(41) $$x_{i}(t+1)=x_{i}(t)+v_{i}(t+1)$$

where *v_i_*(*t*) is the velocity of particle *i* at time *t*. *x_i_*(*t*) is the position of the particle. *w* is the inertia weight. *c*_1_ and *c*_2_ are acceleration coefficients. *r*_1_ and *r*_2_ are random numbers between 0 and 1. *p* is the personal best position of the particle. *g* is the global best position of the swarm. The inertia weight *w* determines the extent to which the particle retains its previous velocity. A larger inertia weight facilitates global exploration, while a smaller inertia weight aids in local exploitation. The acceleration coefficients *c*_1_ and *c*_2_ are known as cognitive and social parameters, respectively, balancing the influence of individual and swarm experiences.

The PSO optimization process involves the following steps. (1) Initialization: randomly initialize the positions and velocities of particles in the search space and evaluate each particle’s fitness value. (2) Update personal best position: if a particle’s current fitness value is better than its historical best position, update its personal best position *p_i_*. (3) Update global best position: if a particle’s current fitness value is better than the current global best position, update the global best position. (4) Update velocity and position: update each particle’s velocity and position according to the velocity and position update formulas. (5) Iteration: repeat steps 2–4 until the maximum number of iterations is reached or the fitness value converges.

In machine learning model optimization, PSO is used to search for the optimal combination of parameters, such as learning rate, regularization parameters, and maximum depth, to minimize the prediction error. The optimization objective can be expressed as follows:

(42) $$\min\sum_{i=1}^{n}l(y_{i},{\hat{y}}_{i})+\Omega (\theta_{k})$$

where *l* is the loss function. ${\hat{y}}_{i}$ is the predicted value. *y_i_* is the actual value. Ω(*θ_k_*) is the regularization term with model parameters *θ_k_*. λ is the regularization parameter.

During the optimization process, the fitness value of each particle is determined by the objective function, which can be the prediction error of the machine learning model. Using PSO to optimize machine learning models can significantly improve model performance and generalization ability. In practice, PSO combined with cross-validation techniques is used to evaluate model performance, and parameter ranges, which are initially set by grid search and random search, are optimized.

In this study, PSO was chosen to optimize the parameters of machine learning models due to its excellent performance in high-dimensional and multimodal optimization problems. The PSO-optimized models can more accurately predict fatigue life and provide reliable regression analysis results.

### 3.3. Model Evaluation Criteria

To comprehensively assess the performance of the fatigue life prediction model across different strategies, multiple metrics are employed: the coefficient of determination (*R*^2^), root mean square error (*RMSE*), and mean absolute percentage error (*MAPE*). *R*^2^ values approaching 1 indicate a high degree of similarity between the predicted and observed values, signifying greater model accuracy. *RMSE* quantifies the deviation between predicted and observed values, with low *RMSE* values reflecting good predictive performance. Due to its sensitivity to large values, *RMSE* can be influenced by outliers. *MAPE* is the average percentage deviation between predicted and observed values. By normalizing the errors of each prediction, *MAPE* offers robust insights into model accuracy, with low *MAPE* values indicating high prediction precision. The formulas for calculating *R*^2^, *RMSE*, and *MAPE* are presented in Equations (43)–(45), respectively.

(43) $$R^{2}=1-\frac{\sum_{n}^{i=1}{\left(y_{i}-{\hat{y}}_{i}\right)}^{2}}{\sum_{n}^{i=1}{\left(y_{i}-\bar{y}\right)}^{2}}$$

(44) $$RMSE=\sqrt{\frac{\sum_{i=1}^{n}{\left(y_{i}-{\hat{y}}_{i}\right)}^{2}}{n}}$$

(45) $$MAPE=\frac{1}{n}\sum_{n}^{i=1}\left|\frac{y_{i}-{\hat{y}}_{i}}{y_{i}}\right|\times 100\%$$

where *y_i_* represents the observed values. ${\hat{y}}_{i}$ represents the predicted values. $\bar{y}$ is the mean of the observed values. *n* is the number of samples.

### 3.4. Model Integration Strategy

**Figure 6:**
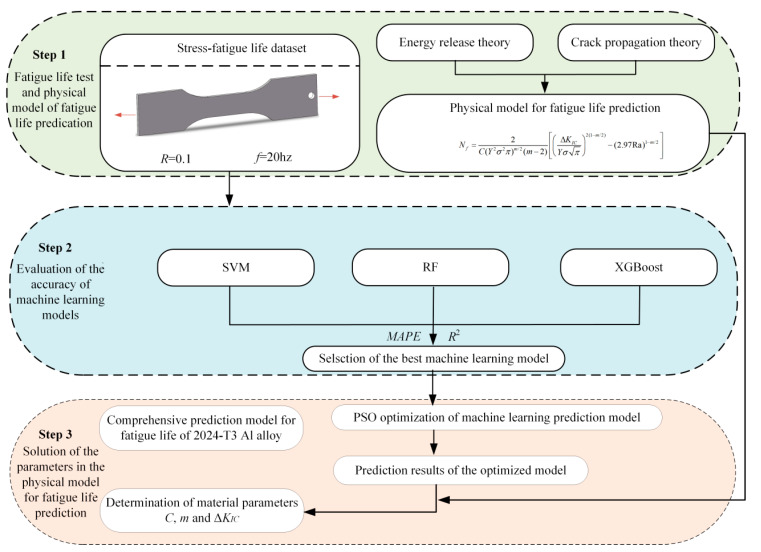
Strategy diagram of fatigue life prediction for Al alloy.

This study integrates physical models with machine learning techniques to develop a fatigue life prediction model for the 2024-T3 Al alloy. The strategy for fatigue life prediction is illustrated in Figure 6. The fatigue life data for the Al alloy are initially obtained under the eight cyclic stresses. Experimental data are used to train the machine learning model for fatigue life prediction. The PSO algorithm is then combined with the machine learning model that has the best predictive performance to enhance accuracy. The predicted output data from the model are used to fine-tune key parameters in the physical model. Thus, this combined approach optimizes the parameters of the physical model, as derived in Equation (14). Notably, *C*, *m*, and $\Delta K_{IC}$ are material constants. The parameters are precisely adjusted through the optimization algorithm, ultimately yielding an accurate physical model.

The implementation of the fatigue life prediction models in this study was carried out using various Python-based libraries. Scikit-learn was employed for the development of traditional machine learning models such as SVM and RF. The XGBoost model was implemented using the XGBoost library. PSO was realized through the Pyswarm library. The computing environment consisted of an Intel Core i7-9700K processor (8 cores, 3.6 GHz), 32 GB of RAM, and an Ubuntu 20.04 operating system. The Python version used was 3.9.

## 4. Results and Discussion

### 4.1. Fatigue Life Prediction Results and Discussion

**Figure 7:**
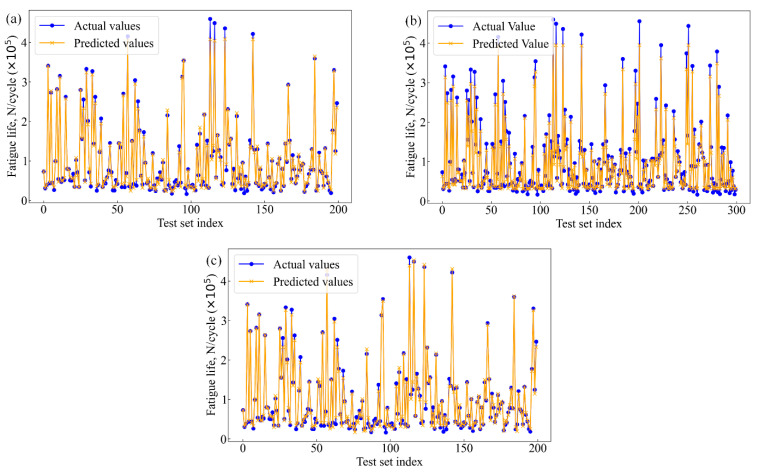
Comparison of actual and predicted values, (**a**) RF, (**b**) SVM, and (**c**) XGBoost.

The fatigue life of the 2024-T3 Al alloy was diagnosed using SVM, XGBoost, and RF models. Figure 7 shows the performance of the machine learning models in predicting fatigue life. The difference between the actual fatigue life values from the test set and the predicted values from each model was compared. Hence, the prediction accuracy of each model and its ability to handle complex nonlinear data are evaluated. As shown in Figure 7, the horizontal axis represents the index of test set specimens, while the vertical axis represents fatigue life. In addition, the blue dots represent the actual fatigue life values measured experimentally, and the yellow lines represent the predicted values. Notably, the overlap between the blue dots and the yellow lines shows the prediction accuracy of each model. The more overlap, the more accurate the prediction. On the contrary, points with a low overlap or noticeable deviation indicate a prediction error.

Figure 7a shows the prediction accuracy of the RF model. This model can capture some basic patterns in the data. However, significant deviations are present in the regions of high cycle fatigue, particularly at certain extreme points. The results indicate that the RF model may lack sufficient generalization ability when dealing with complex nonlinear data, leading to difficulties in accurately capturing finer variations. In general, the RF model is essentially an ensemble learning method based on multiple decision trees. However, the presence of noisy data or extreme values limits its generalization and resistance to noise. For SVM, complex decision boundaries in high-dimensional feature space can be constructed. However, in terms of fatigue life prediction, the SVM model produces more non-overlapping points, as shown in Figure 7b. The prediction accuracy of the SVM model is also poor. This may be attributed to overfitting or underfitting when high-dimensional complex data are handled. Especially for a large number of features and nonlinear data distributions, the prediction accuracy of the SVM decreases. Compared to RF and SVM, most actual and predicted points overlap present in the fatigue life prediction of the XGBoost model, as shown in Figure 7c. The results show that the XGBoost model exhibits high prediction accuracy of the fatigue life. XGBoost employs an ensemble of decision trees based on gradient boosting, which provides strong nonlinear fitting capabilities and efficiently handles noisy data and feature importance evaluation. The excellent generalization ability of the XGBoost model is reflected not only in its adaptability to the training set but also in its robust prediction performance on the test set.

**Figure 8:**
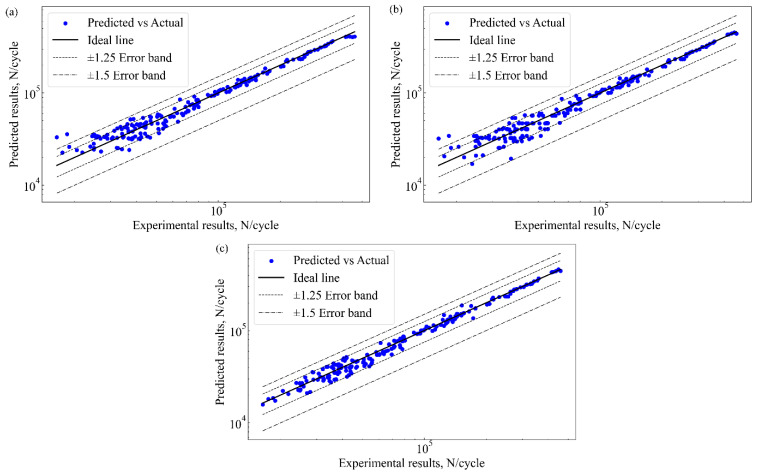
Fatigue life predictions vs experimental results, (**a**) RF, (**b**) SVM, and (**c**) XGBoost.

Figure 8 shows the quantitative relationship between the predicted and actual values of the 2024-T3 Al alloy. Error bands are used to visually display the prediction deviations of each model. The horizontal axis represents the experimentally measured fatigue life values, while the vertical axis shows the predicted results from each model. Each image contains three important lines. The first line is an ideal line that is perfectly aligned between predicted and actual values. The second line is the ±1.25 error band. The third line is ±1.5 error band. Ideally, all data points should fall close to the ideal line. If data points fall within the error bands, the model exhibits high prediction accuracy at that point. However, points outside the error bands indicate large prediction errors. Figure 8a shows that most predicted values of the RF model fall within the ±1.5 error band. The results indicate that the error of the RF model is large. Notably, extreme value predictions are larger. Figure 8b shows the most predicted points within the ±1.5 error band. The limitation of parameter tuning and the nonlinear feature distribution in high-dimensional space decreases the accuracy. The results exhibit similar behavior to the RF. For the XGBoost model, all data are concentrated within the ±1.25 error band, as shown in Figure 8c. The results indicate that XGBoost has high prediction accuracy. The advantage of XGBoost is its ability to handle high-dimensional nonlinear data while balancing accuracy and generalization. This makes it a powerful tool for handling complex engineering tasks.

To further quantify the performance of the models, the *R*^2^ and *MAPE* metrics are calculated. The performance indicators in Table 4 provide a detailed quantitative evaluation of each model’s ability to predict fatigue life. The *R*^2^ and *MAPE* of the XGBoost model are 0.93 and 16.34%, respectively. The highest *R*^2^ indicates that the model shows excellent fitting between the predicted and actual fatigue life of the 2024-T3 Al alloy. The *MAPE* of the average error between the predictions and actual values of XGBoost is only 16.34%. The lowest error shows the robustness and reliability of the XGBoost in capturing complex patterns and nonlinear relationships in the data. The *R*^2^ of the RF model is 0.91, which is 2.15% lower than that of XGBoost. In addition, the MAPE of the RF model is 22.34%, which is 6% higher than XGBoost’s. The results indicate that the RF explains 91% of the variance, and its average prediction error is noticeably large. The SVM model performs the worst, with an *R*^2^ of 0.88 and a MAPE of 26.77%. The *R*^2^ of 0.88 shows that SVM only explains 88% of the data variance, which is 5.38% lower than that of the XGBoost and 3.3% lower than that of the RF. In addition, the *MAPE* of the SVM is the highest among the models, 10.43% higher than that of the XGBoost and 4.43% higher than that of the RF. The results indicate significantly large prediction errors. These differences show that XGBoost is better suited for handling complex fatigue life prediction tasks.

XGBoost exhibits significant superiority over SVM and RF in terms of prediction accuracy and stability. To further enhance its performance, the Particle Swarm Optimization (PSO) algorithm is used to optimize XGBoost’s hyperparameters. The goal of this process is to find better parameter configurations, which could improve the model’s generalization ability and prediction accuracy. Key parameters of the XGBoost’s hyperparameters included the number of estimators, learning rate, maximum tree depth, and minimum child weight. The low fitness value indicates better model performance. To mitigate the risk of overfitting to the validation set during hyperparameter optimization, *k*-fold cross-validation (*k* = 5) was employed to compute the fitness value in each iteration, ensuring the generalization capability of the optimization process. In each iteration of PSO, the training data was split into five subsets. During each iteration, four subsets were used for model training, while the remaining subset was used for validation. This process was repeated five times so that each subset served as the validation set once. For each particle generated in a PSO generation, the fitness value was computed across the five folds. The average fitness value across these five folds was then calculated and used as the generation’s average fitness. The best fitness value among the folds was recorded as the minimum fitness for that generation.

**Figure 9:**
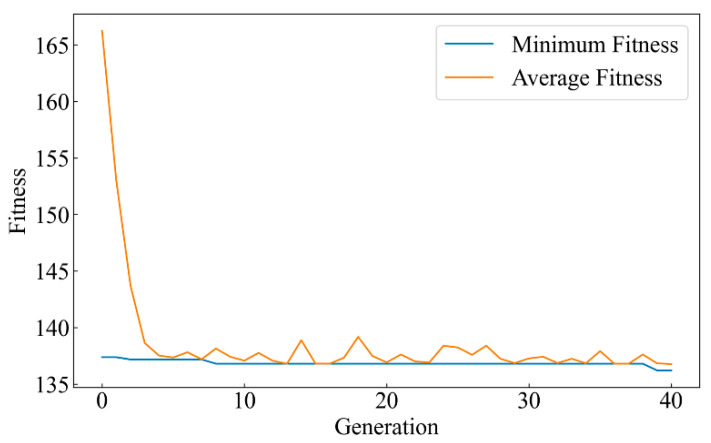
Fitness evolution of XGBoost hyperparameter tuning using Particle Swarm Optimization.

Figure 9 exhibits the fitness curve during the PSO’s optimization of XGBoost’s hyperparameters. The curves show how the model’s performance evolves over the generations (iterations). PSO randomly initializes a group of particles to explore the hyperparameter space, searching for the optimal parameter configuration to improve the model’s performance. The horizontal axis represents the number of iterations, while the vertical axis shows the fitness values. The minimum fitness (blue curve) and average fitness (orange curve) reveal the changes in local and global solutions during each generation.

In the early stages of optimization (the first five iterations), the average fitness value rapidly drops from about 165 to around 140. This indicates that PSO extensively explored the hyperparameter space in the initial stage. The steep decline suggests that PSO quickly identified a set of relatively good hyperparameter combinations, which significantly improved the fitness of the XGBoost model. After the fifth iteration, the decline in fitness slows, and the values begin to stabilize. Notably, the minimum fitness remains around 135, suggesting that PSO is approaching the global optimal solution. In the later iterations, the average fitness shows slight fluctuations but generally remains around 140. These fluctuations reflect the fine-tuning process of PSO in the local search space to further optimize the hyperparameters and get as close as possible to the global optimum. The convergence trend of the fitness curve indicates that PSO effectively completes the optimization of XGBoost’s hyperparameters after about 40 iterations.

The best hyperparameter configuration obtained through PSO optimization is as follows. The number of estimators is set to 1235. This high number of trees helps strengthen the decision boundaries of the model and improves its ability to capture complex data patterns. Then, the learning rate is finely tuned to 0.0905. This relatively low learning rate helps prevent the model from overshooting the optimal solution during training and ensures stable convergence. Additionally, the maximum tree depth is set to 3.00. This relatively shallow depth helps control the model’s complexity and avoids overfitting while still maintaining sufficient performance through the ensemble of many trees. Finally, the minimum child weight was set to 1.00, ensuring that each node split includes a sufficient number of samples, thus improving the model’s robustness, especially when dealing with imbalanced data.

**Figure 10:**
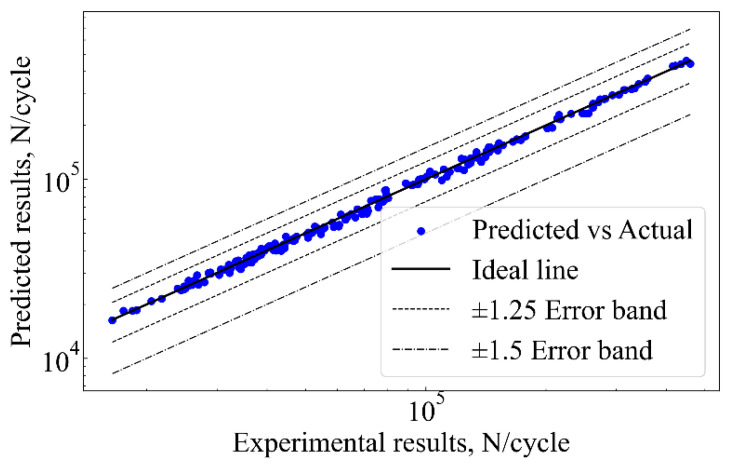
Fatigue life predictions vs. experimental results for PSO-XGBoost.

**Figure 11:**
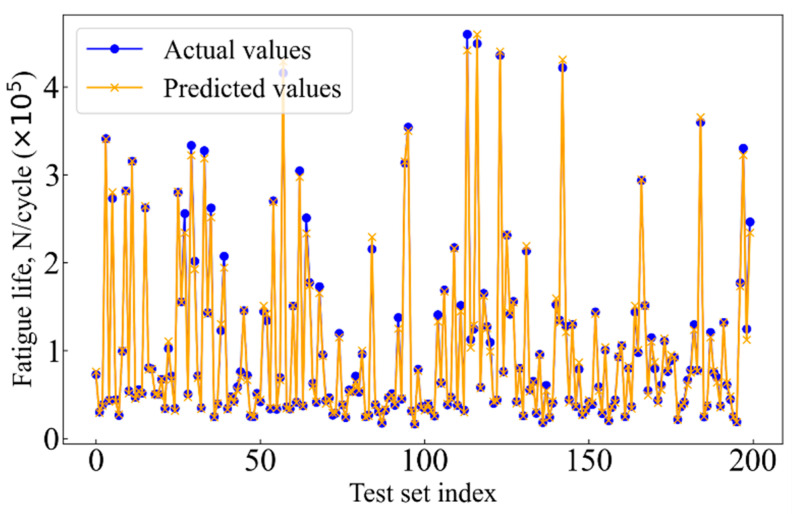
Comparison of actual and predicted values for PSO-XGBoost.

After using PSO to optimize the hyperparameters of the XGBoost model, the PSO-XGBoost model is obtained. The PSO-XGBoost model is then trained for fault diagnosis. Figure 10 presents a plot comparing the predicted and experimental results for the fatigue life of the 2024-T3 Al alloy. The points are plotted against the ideal 45-degree line, which represents perfect agreement between predicted and actual values. Compared to the XGBoost model, the points for the PSO-XGBoost model show a much closer alignment with the ideal line, particularly within the ±1.25 and ±1.5 error bands. This indicates a marked improvement in prediction accuracy. The reduction in scatter and tighter clustering of points near the ideal line suggests that the PSO-XGBoost model has effectively decreased prediction errors and has achieved better generalization. Figure 11 further supports this conclusion by comparing the predicted and actual fatigue life on a logarithmic scale. The PSO-XGBoost model shows a significantly greater overlap between predicted and actual values across the entire test set. This overlap confirms the model’s improved capacity to capture the nonlinear relationships in the data, especially for specimens with extreme or highly variable fatigue life. The orange lines representing predicted values closely follow the blue dots of the actual values, indicating a strong correlation and high prediction accuracy.

The performance of the model, based on two key metrics, is quantitatively analyzed. The *R*^2^ and *MAPE* are listed in Table 5. For the XGBoost model, the *R*^2^ value is 0.93, while the *MAPE* is 16.34%, reflecting good performance in predicting fatigue life. However, after PSO optimization, the PSO-XGBoost model achieves an *R*^2^ of 0.96, showing a 3% improvement in the variance explained by the model. The *MAPE* drops to 11.89%, reflecting a substantial reduction in the average prediction error. This improvement demonstrates that PSO’s global hyperparameter search significantly enhances the model’s predictive accuracy by fine-tuning parameters such as the number of estimators, learning rate, and tree depth.

As shown in Figure 10 and Figure 11, as well as Table 4, the PSO-XGBoost model outperforms the XGBoost, RF, and SVM models in all key evaluation metrics. The PSO-XGBoost model is able to closely match the predicted values with the actual experimental results, which highlights its excellent accuracy and robustness.

### 4.2. Physical Model Parameter Optimization

**Figure 12:**
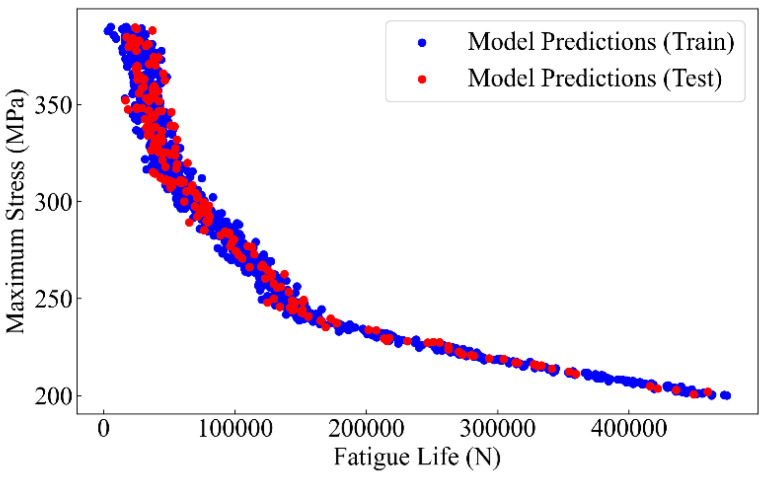
Fatigue life prediction using PSO-XGBoost model.

The PSO-XGBoost model is identified as the optimal choice for predicting the fatigue life of the 2024-T3 Al alloy. Then, the parameters *C*, *m*, and Δ*K*_IC_ in the physical model are determined using the predictive values of the model. The PSO-XGBoost model is first employed to predict the fatigue life of the 2024-T3 Al alloy, and the S-N curve is subsequently plotted based on the predicted values, as shown in Figure 12.

The L-BFGS-B (Limited-memory Broyden–Fletcher–Goldfarb–Shanno with Box constraints) algorithm is employed to optimize the parameters of the physical model. The L-BFGS-B algorithm is a numerical method suitable for large-scale optimization problems [44]. It uses limited memory and gradient information to approximate the Hessian matrix, achieving efficient parameter optimization. This algorithm not only considers parameter boundary constraints but also effectively handles high-dimensional problems. The application of the L-BFGS-B algorithm to Equation (15) contributes to the optimal values for the parameters *C*, *m*, and Δ*K*_IC_, which are determined to be 1.44 × 10^−10^, 2.60, and 36.5 MPa·m^1/2^, respectively. For the 2024-T3 Al alloy, according to the literature and experimental data, the value of parameter *C* is typically in the order of from 10^−10^ to 10^−9^. The parameter *m*, which describes the sensitivity of the crack growth rate to changes in the stress intensity factor range, generally falls between 2 and 4 [45]. The parameter Δ*K*_IC_ of the 2024-T3 Al alloy generally ranges between 35 MPa·m^1/2^ and 40 MPa·m^1/2^ [46]. Therefore, the parameter values obtained in this study are both accurate and reasonable. Furthermore, the parameter solving method for the physical model of fatigue life prediction of the 2024-T3 Al alloy proposed in this study is accurate. In addition, the fatigue life prediction equation for the 2024-T3 aero Al alloy considering different surface roughness is ultimately obtained.

## 5. Conclusions

(1) A physical model was established using the energy method of fracture mechanics. Based on the fatigue fracture characteristics of the 2024-T3 Al alloy, the failure mechanism under the coupling effect of dislocation slip and surface roughness was revealed. Then, the fatigue life prediction equation was established by considering the energy changes during the fatigue crack initiation and propagation. The parameters of the equation include material constants and fracture toughness.
(2) The combination of PSO and XGBoost improved the prediction accuracy of the fatigue life of the 2024-T3 Al alloy. By analyzing the accuracy of RF, SVM, and XGBoost in the fatigue life prediction, it is found that the XG-Boost possesses a high *R*^2^ and low MAPE. Thus, the XGBoost model was selected to predict the fatigue life. Subsequently, the PSO algorithm was employed to optimize the hyperparameters of the XG-Boost model, resulting in improved prediction accuracy.
(3) A physical equation for predicting the fatigue life of the 2024-T3 Al alloy was proposed. Using the fatigue life predictions from the PSO-XGBoost model, the key parameters of the physical fatigue life prediction model were determined. The values of the parameters align with existing experimental data for the 2024-T3 Al alloy. This implied that the physical model of fatigue life proposed in this study is reasonable.

This study established a physical model for fatigue life based on the physical relationship between surface roughness and initial crack length. Considering factors such as the shape and surface defects of structural components, an equivalent physical relationship between the factors and the initial crack length is established. Then, using the fatigue life prediction method proposed in this study, the fatigue life of metallic structural components in the equipment manufacturing industry can be accurately predicted.

## Figures and Tables

**Table 1:** Chemical composition of 2024-T3 Al alloy (wt.%).

Cu	Si	Fe	Mn	Mg	Zn	Cr	Ti	Other	Al
3.8~4.9	0.5	0.5	0.3~0.9	1.2~1.8	0.25	0.1	0.15	0.15	Other

**Table 2:** Mechanical properties of 2024-T3 Al alloy.

Elastic Modulus E (GPa)	Tensile Strength σ_b_ (MPa)	Yield Strength σ_S_ (MPa)	Elongation δ (%)
74.0	466	333	22.8

**Table 3:** Statistical analysis of fatigue life.

Minimum (N)	Maximum (N)	Mean (N)	Median (N)	Standard Deviation (N)
15,161.00	827,501.00	141,953.15	76,693.00	174,688.49

**Table 4:** Comparison of prediction accuracy for different models.

ML Model	*R* ^2^	*MAPE* [%]
RF	0.91	22.34
SVM	0.88	26.77
XGBoost	0.93	16.34

**Table 5:** The results of XGBoost and PSO-XGBoost.

ML Model	*R* ^2^	*MAPE* [%]
XGBoost	0.93	16.34
PSO-XGBoost	0.96	11.89

## Data Availability

The original contributions presented in the study are included in the article, further inquiries can be directed to the corresponding authors.

## Nomenclature

XGBoost	Extreme Gradient Boosting	*N_f_*	Fatigue Life
SVM	Support Vector Machine	*G*	Energy Release Rate
RF	Random Forest	*R* ^2^	Coefficient of Determination
PSO	Particle Swarm Optimization	*RMSE*	Root Mean Square Error
SEM	Scanning Electron Microscope	*MAPE*	Mean Absolute Percentage Error
Ra	Surface Roughness	*σ*_max_	Maximum Cyclic Stress
E	Elastic Modulus	*a*	Crack Length
*σ* *_b_*	Tensile Strength	Δ*K*	Stress Intensity Factor
*σ* *_S_*	Yield Strength	*Y*	Shape Factor
Δ*K_IC_*	Stress Intensity Factor Range	*k_t_*	Stress Concentration Coefficient
